# Recent novelties in research and management of cerebrospinal cavernous malformations

**DOI:** 10.1007/s00701-024-06378-3

**Published:** 2024-11-30

**Authors:** Laurèl Rauschenbach, Philipp Dammann, Ulrich Sure

**Affiliations:** 1 Department of Neurosurgery and Spine Surgery, University Hospital Essen, Essen, Germany; 2 Center for Translational Neuroscience and Behavioral Science (C-TNBS), University of Duisburg-Essen, Essen, Germany; 3 DKFZ Division of Translational Neurooncology at the West German Cancer Center (WTZ), German Cancer Consortium (DKTK), partner site Essen/Düsseldorf, a partnership between DKFZ and University Hospital Essen, University Duisburg-Essen, Essen, Germany

**Keywords:** Cavernous malformation, Central nervous system, Treatment, Surgery

## Abstract

In recent years, knowledge about cerebrospinal cavernomas has grown considerably, leading to the development of initial guidelines and treatment recommendations. However, due to the rarity and heterogeneity of the disease, the level of evidence remains limited, leaving many questions unanswered and subject to ongoing debate. Therefore, an up-to-date review of this field's latest developments and controversies is reasonable.

## Introduction

Cavernous malformations (CMs) of the central nervous system (CNS), also known as cavernomas, cavernous angiomas, or cavernous hemangiomas, are a distinct type of neurovascular lesion characterized by clusters of abnormally dilated capillaries with thin walls that lack intervening brain parenchyma [8]. Sporadic CMs are often associated with developmental venous anomalies (DVAs), which are benign vascular malformations marked by an atypical venous arrangement in the brain [86]. CMs are predisposed to hemorrhagic events and may rarely exhibit neoplastic-like behavior, leading to their inclusion in the World Health Organization (WHO) classification of CNS tumors [52].

**Fig. 1:**
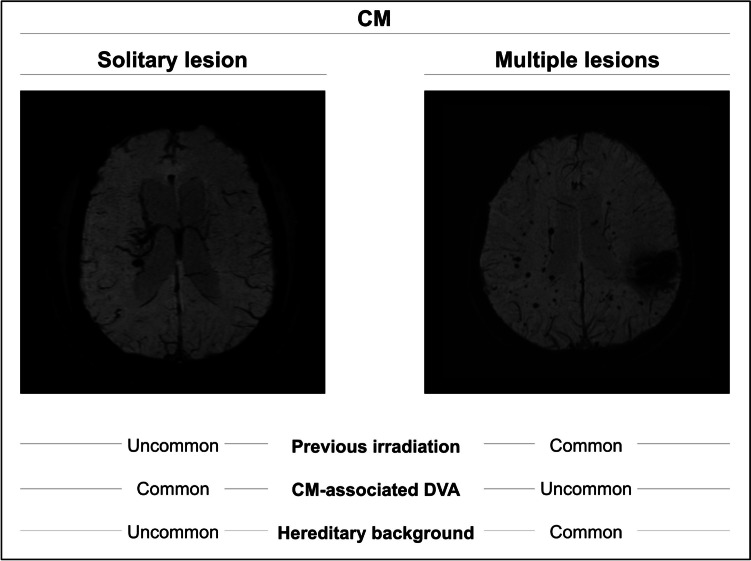
Comparison of the clinical and imaging characteristics of solitary and multiple lesions. Shown are two exemplary, axially-layered MRI-SWI sequences: one from a patient with a solitary lesion and associated DVA (left) and another from a patient with multiple lesions without an associated DVA (right). While multiple lesions are often attributable to previous irradiation or a hereditary origin and are less frequently associated with a DVA, the opposite is true for solitary lesions. This allows for an initial assessment of the underlying etiology of the condition, though definitive conclusions cannot be drawn, as exceptions exist on both sides

CMs may occur sporadically or as part of a familial syndrome. Sporadic CMs typically present as solitary lesions but may occasionally appear as multiple lesions, especially after previous whole-brain irradiation in infancy or when associated with large DVAs. Familial CMs are inherited in an autosomal dominant pattern and are characterized by multiple lesions resulting from germline loss-of-function mutations in the CCM1 (KRIT1), CCM2 (MGC4607), and CCM3 (PDCD10) genes [80]. When genetic testing is unavailable, the presence of both CMs and DVAs may help differentiate the sporadic from familial forms. In individuals who have not previously undergone irradiation, the presence of a DVA alongside one CM typically suggests a sporadic origin, whereas multiple CMs without a DVA are more indicative of a familial form. Clinically, patients with a solitary lesion, an associated DVA, and no family history of affected relatives are very likely to have a sporadic disease, even in the absence of genetic testing [62]. Figure 1 attempts to illustrate the distinguishing characteristics of sporadic and multiple CMs. DVAs are found incidentally in about 3% of the population [56]. While they typically do not carry a significant risk of hemorrhage, these lesions are believed to be a contributing factor in the development of CMs in their vicinity, which may occur in up to 7% of patients over their lifetime [10]. Current guidelines recommend genetic testing in patients with multiple CMs when there is no associated DVA, history of brain irradiation, or a positive family history [2].

The clinical presentation of CMs varies widely, ranging from asymptomatic cases to those with seizures or neurological deficits caused by a symptomatic hemorrhage or mass effect. This variability, combined with the significant impact of lesion localization has led to substantial efforts to differentiate which patients require immediate treatment and which may benefit from a conservative, watch-and-wait approach. Surgical resection remains the primary treatment for symptomatic or high-risk CMs, aiming to completely remove the lesion, minimize neurological deficits, and prevent future hemorrhages [46]. Advances in imaging, microsurgical techniques, and intraoperative monitoring have significantly improved surgical outcomes, reducing the risk of iatrogenic morbidity. For patients not eligible for surgery, non-surgical options, such as antiepileptic drugs, offer alternative treatments. Furthermore, ongoing research on stereotactic radiosurgery and pharmacological interventions seeks to identify therapies that may reduce hemorrhage risk or modify the disease course.

This narrative review is based on long-term clinical experience from a high-volume referral center for sporadic and familial CMs. It aims to provide an updated synthesis of current strategies and treatment indications for CNS-CMs, drawing on recent literature and the insights gained from our clinical practice. To address the considerable heterogeneity of the disease, we will examine CMs in different anatomical regions of the CNS, including supratentorial and cerebellar, brainstem, and spinal cord lesions. By integrating recent advances and current best practices, we hope to guide clinicians toward state-of-the-art treatments and facilitate patient-centered decision-making based on the latest guidelines, ultimately improving outcomes and quality of life for those affected by this complex condition.

## General considerations in CNS-CM surgery

The treatment options for CNS-CMs range from conservative approaches to surgical interventions, depending on the characteristics of the lesions and the clinical profile of the patients. Although debated, surgical resection remains the gold standard and the only curative option for symptomatic or high-risk CMs with a history of hemorrhage. However, the decision to proceed with surgery often requires careful consideration of the natural history of the disease versus the potential risks of surgery, since the risk of postoperative complications may be equal to or greater than the risk of leaving the lesion untreated [58]. Various factors, both objective and subjective, influence the appropriateness of surgery, including anatomic features (e.g., lesion size and localization), clinical presentation (e.g., symptoms, severity of neurological deficits, number and frequency of bleeding events, and presence of familial cavernomatosis), and patient-specific factors (e.g., age, life expectancy, comorbidities, and personal fears related to surgery, unexpected bleeding events, or sudden unexpected death in epilepsy cases). Randomized controlled trials comparing surgical and conservative treatments are scarce [69], and most available evidence comes from observational studies, which are often limited by selection bias, single-center recruitment, and short follow-up periods [63, 82]. Despite these limitations, recent efforts to conduct higher-quality research studies have been encouraging, though definitive results are still pending [1, 6]. In the meantime, clinical decisions can be guided by existing resources such as the 2013 guidelines from the International League Against Epilepsy [70] and the 2017 guidelines from the Angioma Alliance [2], as well as expert opinions derived from consensus processes [16, 81].

The following general surgical considerations are important in CM surgery:

1. **Symptomatology**: CMs may cause symptoms through their mass effect or more commonly as a result of hemorrhage, which can exacerbate pre-existing symptoms. Hemorrhagic events should be clearly defined as the acute or subacute onset of neurological symptoms (such as headache, seizure, impaired consciousness, or focal neurological deficits) that can be linked to the anatomical location of a CM and confirmed by imaging [4]. Asymptomatic growth, hemorrhage, or the detection of perifocal hemosiderin deposits alone do not meet the criteria for a hemorrhagic event. This definition helps explain why brainstem and intramedullary CMs are often perceived as more aggressive. Their perceived aggressiveness is likely due to their location in highly eloquent CNS structures, where even small hemorrhages can lead to significant neurological deficits. In rare cases, CMs can cause cluster hemorrhages, characterized by recurrent bleeding episodes occurring in rapid succession, leading to rapid neurological deterioration [16].
2. **Risk of hemorrhage**: Several studies have evaluated the natural history of CMs and identified risk factors for bleeding. Patients who have experienced a prior hemorrhage are at significantly higher risk for future hemorrhages than those in whom the CM was discovered incidentally [5, 41]. This supports the rationale for a conservative, watch-and-wait approach in asymptomatic patients and favors treatment initiation after an initial or recurrent bleeding event. Our own studies suggest that obese patients and those receiving female hormone treatment may also have an increased risk of experiencing hemorrhages [14, 92].
3. **Surgical advances**: Recent advances in microsurgical techniques, including neuronavigation [89], imaging [11], and electrophysiological monitoring [51], have enhanced the precision and safety of CM removal. Surgeons now have access to advanced preoperative imaging techniques such as diffusion-tensor MRI with tractography [3], navigated transcranial magnetic stimulation [60], and task-based functional MRI [90]. Depending on the lesion location this can be crucial for preoperative planning, as white matter tracts may be displaced by the CM but can still pass through the adjacent hemosiderin ring, which may be partially resected [12]. For cases involving epilepsy, detailed preoperative epileptological diagnostics, such as video EEG monitoring, are useful [75]. Intraoperative tools such as microsurgical techniques [20, 47], electrophysiological monitoring (cortical and subcortical stimulation) [51, 66], frameless image-guided neuronavigation [89], neuro-navigated ultrasonography [25], and, in certain cases, awake surgery [61], further contribute to minimal surgical morbidity.
4. **Extent of resection**: Achieving complete lesion removal is crucial for preventing recurrent hemorrhages and ongoing seizures [45]. Debate exists regarding the optimal surgical approach, whether transsulcal [38] or transparenchymal [47], but most agree that a circumferential, intracapsular approach with en bloc resection provides the best results in supratentorial CM surgery. Resection is typically most effective several weeks after a hemorrhage, when the lesion has clearer delineation from surrounding brain tissue and perifocal edema has resolved, allowing for safer and more effective excision [26]. For lesions in deep or eloquent areas, central debulking followed by piecemeal resection is often necessary and feasible, as CMs lack arterial input. Meticulous care must be taken to avoid leaving residual CM tissue, as complete resection is crucial for surgical success.
5. Preserving **DVAs**: Preservation of DVAs during surgery is important, as these vessels provide normal and usually critical venous drainage for the affected individual. Disruption of a DVA increases the risk of venous thrombosis and infarction, potentially leading to secondary hemorrhage. Some authors propose removing the distal branches of the DVA while preserving the main trunk to prevent CM recurrence, but this approach remains under-researched [88]. Available guidelines and surveys, however, recommend preserving DVAs to avoid secondary complications [2, 16, 81].
6. **Epilepsy**: In cases of CM-related epilepsy (CRE), the epileptogenic focus can extend beyond the CM itself. In patients with medically refractory seizures, resection should aim to include the hemosiderin-enriched gliotic tissue (if this area is not eloquent) surrounding the CM, as this tissue may also be a source of seizure activity [71]. Removing hemosiderin-enriched tissue is less significant when CMs are resected early in the course of epilepsy, which is generally recommended [28].
7. **Case load**: For deep or eloquently located CMs, surgery should be performed in high-volume centers with neurovascular experience. Evidence suggests that hospitals with higher case volumes are associated with better neurological outcomes [72], a trend observed in other neurovascular conditions [21]. The rarity of the disease and the complexity of decision-making highlight the importance of treatment in specialized centers with multidisciplinary teams.
8. **Quality of life**: Patients with CMs may experience a significant decline in quality of life following diagnosis, symptomatic hemorrhage, or surgical intervention [17, 39, 65, 77]. This factor is frequently overlooked when the focus is primarily on focal neurological deficits. However, quality of life considerations should play a central role in counseling affected individuals. These factors should also be taken into account during the final treatment decision-making process and in measuring outcomes throughout therapy.

For patients who are not suitable candidates for surgery—such as those with asymptomatic CMs, no history of hemorrhage, or lesions in high-risk locations—conservative management is recommended [2]. Notably, no formal guidelines exist on the necessity or frequency of imaging for conservatively managed patients, emphasizing the need for individualized care based on clinical judgment. We currently recommend regular follow-up MRI examinations for asymptomatic lesions every 3–5 years. However, immediate imaging is essential if new symptoms occur. Radiosurgery is considered a second-line treatment in sporadic cases, though its direct impact on bleeding risk remains controversial [23, 64]. In familial cases, radiosurgery is generally discouraged, as it may lead to the development of new lesions [2]. Antiepileptic drugs may be used in cases of CM-associated epilepsy as part of the conservative management plan. Endovascular treatment has no role in CM therapy.

## Surgical treatment of sporadic supratentorial and cerebellar malformations

For superficial and non-eloquent CMs, surgical risks are generally low, as these lesions tend to be accessible with minimal risk of morbidity. Several clinical conditions justify surgical resection. There is no definitive consensus among experts regarding the resection of asymptomatic lesions in either children or adults [81]. However, potential benefits include preventing future hemorrhage, alleviating the psychological burden of living with a potentially hemorrhagic lesion, and reducing the need for long-term follow-up. Moreover, resection may help facilitate lifestyle or career decisions by eliminating the lesion as a future risk [2]. In our clinical practice, we typically refrain from excising asymptomatic lesions due to the relatively low risk of an initial symptomatic hemorrhage. Surgery, however, should be considered in symptomatic patients to relieve the mass effect, improve neurological function, and prevent recurrent hemorrhages [2]. The indication for surgery is very strong in patients with CRE when seizures are medically resistant, and there is compelling evidence linking the solitary CM to the epileptic focus [2, 70]. Exceptions may be made for patients who refuse anticonvulsive therapy, are noncompliant with their medications, or suffer from a CM with a high rate of hemorrhages [70]. The timing of surgical intervention for CRE remains a subject of debate. Increasing evidence suggests a correlation between the duration of epilepsy and postoperative outcomes, with longer durations of epilepsy being linked to less favorable postoperative seizure control [15, 19, 27, 73]. This has triggered discussions on whether earlier surgical intervention should be favored in patients with CRE, as prolonged use of antiepileptic drugs and the constant awareness of a potentially hemorrhagic lesion can significantly impact a patient’s quality of life [65].

In contrast, deep-seated lesions such as those in the basal ganglia, thalamus, or other eloquent brain regions present a greater challenge. Surgery for these CMs carries a higher risk of morbidity due to their proximity to vital structures [87]. As a result, surgical intervention should be limited to patients who are severely symptomatic or who have experienced previous hemorrhage(s). For asymptomatic patients, surgery is generally not recommended. There is also considerable debate regarding the appropriate timing of surgery in these cases, with some experts advocating for resection after the first hemorrhagic event [2], while others recommend delaying surgery until after a second hemorrhage to reduce the risk of iatrogenic injury [81].

Scoring systems have been developed to objectively assess the appropriateness of surgery for deep-seated or eloquently located CMs. These scoring systems, combined with clinical guidelines from the Angioma Alliance, can serve as valuable tools for preoperative planning [29].

## Surgical treatment of sporadic brainstem malformations

Brainstem cavernous malformations (BSCMs) are associated with a high risk of symptomatic hemorrhage and iatrogenic morbidity. The brainstem contains densely packed, highly eloquent nuclei and fiber tracts within a small area, making these lesions particularly challenging to access surgically [54]. For decades, BSCMs were considered a surgical no-man's-land [49]. However, advances in imaging, surgical approaches, and microsurgical techniques have fundamentally changed this view. Today, with careful consideration of surgical indications, these procedures can be performed with favorable outcomes [45].

Surgical intervention in BSCM patients should be reserved for symptomatic lesions. Guidelines do not provide a definitive recommendation on the number of hemorrhagic events that should occur before considering surgery. While some experts consider surgery after the first disabling hemorrhagic event controversial, resection is generally recommended after a second hemorrhagic event [2]. This approach is supported by surveys of experienced neurosurgeons, with the majority favoring resection after a second symptomatic hemorrhage [81].

A comprehensive consensus on BSCMs provides detailed guidance, covering a range of clinical scenarios and influencing factors [16]. The consensus defines hemorrhagic events, management of associated DVAs, handling of postoperative remnants, safe entry zones, and strategies for specific anatomic locations. It also addresses the complexity of BSCMs by distinguishing between lesions that are easily accessible and those that are more difficult to access. For easily accessible brainstem lesions with moderate to severe deficits, resection after the first hemorrhagic event is typically favored. In contrast, for difficult-to-access lesions or those associated with mild deficits, surgery is often postponed until after the second hemorrhagic event.

In addition to consensus guidelines, several scoring systems have been proposed to predict surgical outcomes for BSCMs [18, 31, 85]. These tools can complement the Angioma Alliance guidelines and Delphi Consensus Projects, helping clinicians refine their surgical decision-making process. Most experts recommend surgery 2 to 8 weeks after the most recent hemorrhage, once a dissection membrane has formed, post-hemorrhagic swelling has subsided, and gliosis has not yet developed [45].

## Surgical treatment of sporadic intramedullary malformations

Compared to cerebral CMs, there is relatively limited research on spinal malformations. As a result, the Angioma Alliance guidelines do not provide specific recommendations for spinal CMs beyond suggesting that they be managed similarly to BSCMs due to the high eloquence of the spinal cord [2]. Reviews generally support a conservative approach for asymptomatic spinal CMs, with surgery reserved for patients after a first hemorrhagic event [7, 9, 30]. This aggressive treatment recommendation in symptomatic spinal CMs is justified because of recurrent bleeding and an impaired neurological recovery with each subsequent hemorrhagic event [67]. Surgical decision-making is particularly challenging for difficult-to-reach lesions, such as ventrally located non-exophytic CMs or those in the thoracic or lumbar spinal cord. In these cases, surgery may be postponed until after a second hemorrhagic event, particularly if the patient only has mild neurological deficits [36, 68]. While no expert consensus specifically addresses spinal CMs, there appears to be alignment with BSCM guidelines, with most studies recommending surgery within three months of a hemorrhage [7, 9, 36, 68]. One of the few prospective studies in this field advises caution against early intervention, suggesting that surgery should be delayed until a dissection membrane has formed, similar to BSCMs [43].

## Surgical treatment of CNS-CM multiplicity

Multiple CMs may develop sporadically or as part of familial cavernomatosis. In patients with familial CMs, germline mutations in the CCM1, CCM2, or CCM3 genes result in the continual formation of new lesions, making curative surgery impossible. Thus, surgical treatment in patients with multiple CMs should be limited to symptomatic lesions, following the same criteria used for solitary symptomatic lesions. Asymptomatic lesions should not be treated surgically [16]. Interestingly, studies indicate that patients with multiple CMs do not necessarily have a higher cumulative risk of hemorrhage compared to those with solitary CMs when analyzed over short-term follow-up periods of 5 years [41, 74]. Ongoing research is focused on determining whether patients with CCM3 mutations are at a higher risk for early onset of symptoms and increased incidence of hemorrhage compared to those with CCM1 or CCM2 mutations, though this remains an area of active investigation [22, 76].

## Clinical practice – balancing evidence-based medicine and surgical experience

The current data and recommendations for managing CM patients have greatly improved the quality of care at our center and are well integrated into clinical practice. However, extensive clinical experience remains essential for accurately assessing surgical risks and providing informed patient counseling. At our center, patients attend a dedicated clinic, where they receive comprehensive information about their condition and, if necessary, undergo additional diagnostic evaluations. In our approach, asymptomatic patients are generally not considered for surgery, as the operative risk typically outweighs the low risk of spontaneous bleeding in stable CMs. Exceptions may be made for superficial, non-eloquent lesions if there is a strong and well-justified patient preference for surgery. Patients with a history of hemorrhage, as a prior bleeding event is the most significant predictor of future bleeds, are prioritized for surgical evaluation. For superficial supratentorial and cerebellar CMs with a history of bleeding, we offer surgical resection, usually after the first bleed. CMs located in eloquent or cortical areas require additional preoperative imaging and intraoperative monitoring, such as diffusion-tensor MRI with tractography, intraoperative electrophysiology, or awake surgery, to ensure safe resection. Sometimes, a second symptomatic hemorrhage causes an enlargement of the lesions that may then allow a less invasive dissection for the removal. For patients with CRE, surgery is indicated if they have drug-resistant epilepsy, as defined by the ILAE, i.e. failure to achieve seizure freedom with two antiepileptic drugs. Exceptions are made for patients with previous hemorrhages and those who prefer not to undergo long-term medication. Preoperative evaluation of the epileptogenic focus is always necessary to confirm a clear link between the lesion and epilepsy. Deep supratentorial CMs, such as those in the thalamus or basal ganglia, are assessed based on surgical accessibility, neurological morbidity, and patient preference, with surgery potentially considered after two symptomatic bleeding episodes. BSCMs are not surgically treated if asymptomatic. However, unlike the Angioma Alliance guidelines, we adopt a more individualized approach, sometimes opting for surgery after a single bleed. For accessible, superficially located lesions and in patients with moderate to severe deficits, surgery is considered after the first bleed. In contrast, for deep-seated lesions in patients with mild symptoms, we typically defer surgery until a second bleed. For spinal CMs, we are usually more aggressive compared to BSCMs, with surgery considered after the first symptomatic bleed in accessible lesions, because a second hemorrhage in these patients often causes a new irreversible functional deficit.

Patients with newly occurring or recurrent hemorrhages are typically admitted to the hospital and monitored for several days, depending on their neurological status, either in the intensive care unit, intermediate care unit, or general ward. Once the MRI has been updated and cluster bleeding has been ruled out, these patients are referred for rehabilitation. Patients with an indication for surgery undergo the procedure approximately 2–6 weeks after the latest hemorrhage (especially in eloquent lesions), while those without a surgical indication enter a "watch and wait" phase.

## Concerns and perspectives

Research into cavernous malformations continues to evolve, with an increasing volume of scientific literature contributing valuable insights into the epidemiology, etiology, pathogenesis, natural history, and therapeutic strategies for these lesions. The development of systematic literature reviews has led to the creation of initial treatment guidelines, such as those from the Angioma Alliance in 2017, which have been supplemented by various consensus projects that address unresolved issues in CM management.

However, research into CMs faces significant limitations. The heterogeneity of the disease and the rarity of hemorrhagic events present challenges for designing large, randomized controlled trials, particularly for brainstem surgery. Additionally, existing guidelines and consensus projects may not fully translate into clinical practice, as they are often subject to differing interpretations by proponents of either conservative or surgical approaches. For example, the term "symptomatic" is used broadly in studies to describe a wide range of neurological conditions, from mild dysesthesias to severe motor deficits. Similarly, terms like "easily accessible" or "inoperable" are highly subjective and strongly vary among surgeons. Consequently, the management of CMs remains largely individualized, particularly in smaller neurosurgical centers with a low caseload, though it is increasingly integrated into an evidence-based framework rather than being guided solely by expert opinion.

While surgical treatment has advanced and improved in safety, practical limitations persist. Emerging technologies such as radiosurgery [23, 64] and laser interstitial thermal therapy for CRE cases [53] have been applied in the treatment of CMs, though no clear recommendations exist for their use. At this time, we do not recommend using these treatment options, as the literature partly provides conflicting results, is currently limited to short-term follow-up investigations and both approaches do not finally eliminate the risk of re-hemorrhages. Moreover, radiation-induced CM formation is well documented, even after stereotactic radiosurgery [23, 64]. Finally, studies that focus on CM size as a primary outcome parameter also have limited relevance due to the unpredictable nature of the disease.

Recent interest in drug repositioning—using existing medications such as HMG-CoA reductase inhibitors, platelet aggregation inhibitors, or beta-adrenergic receptor antagonists—has gained momentum, although current evidence is mixed. Regardless of the pending results, current developments in pharmacotherapy testify to the success of evidence-based medicine in CM research, exemplified by HMG-CoA reductase inhibitors. Enabled by the decoding of increased RhoA/ROCK activity in CMs [24], in vitro and in vivo experiments were conducted several years ago, which ultimately progressed from retrospective observational studies to prospective clinical trials [13, 35, 55, 83]. The results of these studies are eagerly awaited, particularly the forthcoming data from the Phase I/II randomized, placebo-controlled, double-blinded AT CASH EPOC trial (NCT02603328) on atorvastatin, which exhibits pleiotropic effects, including RhoA/ROCK inhibition. At present, the therapeutic use of beta-adrenergic receptor antagonists like propranolol [42, 48] and aspirin [59, 91], which have shown a reduction in bleeding rates in retrospective trials, has not yet been incorporated into our clinical routine recommendations. However, phase 3 trials on propranolol and aspirin are anticipated and could significantly impact future management. The recognition that CMs share characteristics with neoplastic structures has redirected research toward the pharmacological modulation of CM-associated signaling pathways [78]. Preclinical studies are investigating therapies targeting pathways such as RhoA/ROCK (e.g., NRL-1049 or Fasudil), mTOR (e.g., Rapamycin), superoxide dismutase (e.g., REC-994), VEGF (e.g., Semaxanib or Sorafenib), MAP (e.g., Ponatinib), and TIE2 (e.g., Rebastinib). These studies have already led to the initiation of early clinical trials, including the randomized, double-blind, placebo-controlled SYNCAMORE trial on the use of REC-994 [57]. Gene therapies, though theoretically promising for addressing dysfunctional genes, are not yet viable treatment options [40, 84].

Furthermore, efforts are underway to identify biomarkers that could predict bleeding events [33]. The advantage of highly sensitive and specific biomarkers would be the ability to monitor disease progression in a way that complements MRI imaging. While this field of research is still in its early stages, a significant number of potential biomarkers have already been identified. These now require further investigation in follow-up studies to assess their clinical applicability [34, 50, 79]. So far, biomarkers have not yet been incorporated into clinical practice, so diagnosis and monitoring continue to rely on neurological examination and imaging diagnostics.

The increasing use of artificial intelligence (AI) across various medical fields suggests that AI will play a significant role in the diagnosis and treatment of neurovascular diseases in the future. This trend is already evident, with a growing body of literature focused on AI-based biomarker discovery for hemorrhage risk prediction [44], identification of targeted therapeutic options [32], and advancements in surgical planning [37]. However, these areas remain subjects of ongoing research and therefore currently do not play a role in our present clinical care.

## Conclusion

Cerebrospinal CMs present a complex clinical challenge due to their unpredictable natural history, variable clinical presentations, and the potential for significant neurological morbidity. Advances in diagnostic imaging, microsurgical techniques, and intraoperative monitoring have improved the outcomes for patients undergoing surgical resection, which remains the cornerstone of treatment for symptomatic or high-risk CMs. However, the decision to intervene surgically must be carefully weighed against the risks and the management of deep-seated or eloquently located lesions remains a subject of debate. Non-surgical treatment, including antiepileptic pharmacotherapy and watchful waiting, is a viable option for patients with asymptomatic or less accessible lesions. Meanwhile, emerging therapies, such as stereotactic radiosurgery and pharmacological approaches targeting specific molecular pathways, may offer future alternatives, although studies are needed to establish their efficacy and safety. The development of evidence-based guidelines and consensus recommendations has provided clinicians with a framework for managing this rare and heterogeneous condition, but the limited availability of high-quality evidence underscores the need for ongoing research.

## Data Availability

No datasets were generated or analysed during the current study.

## Abbreviations

BSCM: Brainstem CM
CRE: CM-related epilepsy
CM: Cavernous malformation
CNS: Central nervous system
DVA: Developmental venous anomaly
WHO: World Health Organization

## References

[1] (2024) Medical management and surgery versus medical management alone for symptomatic cerebral cavernous malformation (CARE): a feasibility study and randomised, open, pragmatic, pilot phase trial. Lancet Neurol 23:565–576. 10.1016/s1474-4422(24)00096-6 10.1016/S1474-4422(24)00096-6 38643777

[2] Akers A, Al-Shahi Salman R, Awad IA, Dahlem K, Flemming K, Hart B, Kim H, Jusue-Torres I, Kondziolka D, Lee C, Morrison L, Rigamonti D, Rebeiz T, Tournier-Lasserve E, Waggoner D, Whitehead K (2017) Synopsis of guidelines for the clinical management of cerebral cavernous malformations: consensus recommendations based on systematic literature review by the angioma alliance scientific advisory board clinical experts panel. Neurosurgery 80:665–680. 10.1093/neuros/nyx091 28387823 10.1093/neuros/nyx091 PMC5808153

[3] Al-Salihi MM, Al-Jebur MS, Al-Salihi Y, Saha R, Daie MM, Rahman MM, Ayyad A (2024) Diffusion tensor imaging with tractography in surgical resection of brainstem cavernous malformations: a systematic review and meta-analysis. Int J Neurosci 134:1075–1097. 10.1080/00207454.2023.2214696 37194114 10.1080/00207454.2023.2214696

[4] Al-Shahi Salman R, Berg MJ, Morrison L, Awad IA (2008) Hemorrhage from cavernous malformations of the brain: definition and reporting standards. Angioma Alliance Scientific Advisory Board. Stroke 39:3222–3230. 10.1161/strokeaha.108.515544 18974380 10.1161/STROKEAHA.108.515544

[5] Al-Shahi Salman R, Hall JM, Horne MA, Moultrie F, Josephson CB, Bhattacharya JJ, Counsell CE, Murray GD, Papanastassiou V, Ritchie V, Roberts RC, Sellar RJ, Warlow CP (2012) Untreated clinical course of cerebral cavernous malformations: a prospective, population-based cohort study. Lancet Neurol 11:217–224. 10.1016/s1474-4422(12)70004-2 22297119 10.1016/S1474-4422(12)70004-2 PMC3282211

[6] Al-Shahi Salman R, Kitchen N, Thomson J, Ganesan V, Mallucci C, Radatz M (2016) Top ten research priorities for brain and spine cavernous malformations. Lancet Neurol 15:354–355. 10.1016/s1474-4422(16)00039-9 26831334 10.1016/S1474-4422(16)00039-9

[7] Asimakidou E, Meszaros LT, Anestis DM, Tsitsopoulos PP (2022) A systematic review on the outcome of intramedullary spinal cord cavernous malformations. Eur Spine J 31:3119–3129. 10.1007/s00586-022-07332-6 35931791 10.1007/s00586-022-07332-6

[8] Awad IA, Robinson JR Jr, Mohanty S, Estes ML (1993) Mixed vascular malformations of the brain: clinical and pathogenetic considerations. Neurosurgery 33:179–188. 10.1227/00006123-199308000-00001. (discussion 188) 8367039 10.1227/00006123-199308000-00001

[9] Badhiwala JH, Farrokhyar F, Alhazzani W, Yarascavitch B, Aref M, Algird A, Murty N, Kachur E, Cenic A, Reddy K, Almenawer SA (2014) Surgical outcomes and natural history of intramedullary spinal cord cavernous malformations: a single-center series and meta-analysis of individual patient data: clinic article. J Neurosurg Spine 21:662–676. 10.3171/2014.6.Spine13949 25062285 10.3171/2014.6.SPINE13949

[10] Brinjikji W, El-Masri AE, Wald JT, Flemming KD, Lanzino G (2017) Prevalence of cerebral cavernous malformations associated with developmental venous anomalies increases with age. Childs Nerv Syst 33:1539–1543. 10.1007/s00381-017-3484-0 28643038 10.1007/s00381-017-3484-0

[11] Campbell PG, Jabbour P, Yadla S, Awad IA (2010) Emerging clinical imaging techniques for cerebral cavernous malformations: a systematic review. Neurosurg Focus 29:E6. 10.3171/2010.5.Focus10120 20809764 10.3171/2010.5.FOCUS10120 PMC3708641

[12] Cauley KA, Andrews T, Gonyea JV, Filippi CG (2010) Magnetic resonance diffusion tensor imaging and tractography of intracranial cavernous malformations: preliminary observations and characterization of the hemosiderin rim. J Neurosurg 112:814–823. 10.3171/2009.8.Jns09586 20367384 10.3171/2009.8.JNS09586

[13] Chen B, Lahl K, Saban D, Lenkeit A, Rauschenbach L, Santos AN, Li Y, Schmidt B, Zhu Y, Jabbarli R, Wrede KH, Kleinschnitz C, Sure U, Dammann P (2022) Effects of medication intake on the risk of hemorrhage in patients with sporadic cerebral cavernous malformations. Front Neurol 13:1010170. 10.3389/fneur.2022.1010170 36686509 10.3389/fneur.2022.1010170 PMC9847255

[14] Chen B, Saban D, Rauscher S, Herten A, Rauschenbach L, Santos A, Li Y, Schmidt B, Zhu Y, Jabbarli R, Wrede KH, Kleinschnitz C, Sure U, Dammann P (2021) Modifiable cardiovascular risk factors in patients with sporadic cerebral cavernous malformations: obesity matters. Stroke 52:1259–1264. 10.1161/strokeaha.120.031569 33588600 10.1161/STROKEAHA.120.031569

[15] Cohen DS, Zubay GP, Goodman RR (1995) Seizure outcome after lesionectomy for cavernous malformations. J Neurosurg 83:237–242. 10.3171/jns.1995.83.2.0237 7616268 10.3171/jns.1995.83.2.0237

[16] Dammann P, Abla AA, Al-Shahi Salman R, Andrade-Barazarte H, Benes V, Cenzato M, Connolly ES, Cornelius JF, Couldwell WT, Sola RG, Gomez-Paz S, Hauck E, Hernesniemi J, Kivelev J, Lanzino G, Macdonald RL, Morcos JJ, Ogilvy CS, Steiger HJ, Steinberg GK, Santos AN, Rauschenbach L, Darkwah Oppong M, Schmidt B, Spetzler RF, Schaller K, Lawton MT, Sure U (2022) Surgical treatment of brainstem cavernous malformations: an international Delphi consensus. J Neurosurg 136:1220–1230. 10.3171/2021.3.Jns2156 34598135 10.3171/2021.3.JNS2156

[17] Dammann P, Herten A, Santos AN, Rauschenbach L, Chen B, Darkwah Oppong M, Schmidt B, Forsting M, Kleinschnitz C, Sure U (2021) Multimodal outcome assessment after surgery for brainstem cavernous malformations. J Neurosurg 135:401–409. 10.3171/2020.6.Jns201823 33065532 10.3171/2020.6.JNS201823

[18] Dammann P, Wrede K, Jabbarli R, Müller O, Mönninghoff C, Forsting M, Sure U (2017) Of bubbles and layers: which cerebral cavernous malformations are most difficult to dissect from surrounding eloquent brain tissue? Neurosurgery 81:498–503. 10.1093/neuros/nyx025 28327920 10.1093/neuros/nyx025

[19] Dammann P, Wrede K, Jabbarli R, Neuschulte S, Menzler K, Zhu Y, Özkan N, Müller O, Forsting M, Rosenow F, Sure U (2017) Outcome after conservative management or surgical treatment for new-onset epilepsy in cerebral cavernous malformation. J Neurosurg 126:1303–1311. 10.3171/2016.4.Jns1661 27367244 10.3171/2016.4.JNS1661

[20] Davies JM, Kim H, Lawton MT (2015) Surgical treatment of cerebral cavernous malformations. J Neurosurg Sci 59:255–270 25881653

[21] Davies JM, Lawton MT (2017) Improved outcomes for patients with cerebrovascular malformations at high-volume centers: the impact of surgeon and hospital volume in the United States, 2000–2009. J Neurosurg 127:69–80. 10.3171/2016.7.Jns15925 27739942 10.3171/2016.7.JNS15925

[22] Denier C, Labauge P, Bergametti F, Marchelli F, Riant F, Arnoult M, Maciazek J, Vicaut E, Brunereau L, Tournier-Lasserve E (2006) Genotype-phenotype correlations in cerebral cavernous malformations patients. Ann Neurol 60:550–556. 10.1002/ana.20947 17041941 10.1002/ana.20947

[23] Dumot C, Mantziaris G, Dayawansa S, Xu Z, Pikis S, Peker S, Samanci Y, Ardor GD, Nabeel AM, Reda WA, Tawadros SR, Abdelkarim K, El-Shehaby AMN, Emad Eldin RM, Elazzazi AH, Moreno NM, Martínez Álvarez R, Liscak R, May J, Mathieu D, Tourigny JN, Tripathi M, Rajput A, Kumar N, Kaur R, Picozzi P, Franzini A, Speckter H, Hernandez W, Brito A, Warnick RE, Alzate J, Kondziolka D, Bowden GN, Patel S, Sheehan J (2024) Stereotactic radiosurgery for haemorrhagic cerebral cavernous malformation: a multi-institutional, retrospective study. Stroke Vasc Neurol 9:221–229. 10.1136/svn-2023-002380 37586775 10.1136/svn-2023-002380 PMC11221296

[24] Eisa-Beygi S, Wen XY, Macdonald RL (2014) A call for rigorous study of statins in resolution of cerebral cavernous malformation pathology. Stroke 45:1859–1861. 10.1161/strokeaha.114.005132 24803598 10.1161/STROKEAHA.114.005132

[25] El-Tantawy IMS, Kassem MA, El-Badry AA, AbdElwahab KM (2022) Intra-operative ultrasound (IOUS) value in cases of AVM and cavernoma excision: single-center experience. Egypt J Neurosurg 37:39. 10.1186/s41984-022-00175-9

[26] Ellis JA, Barrow DL (2017) Supratentorial cavernous malformations. Handb Clin Neurol 143:283–289. 10.1016/b978-0-444-63640-9.00027-8 28552151 10.1016/B978-0-444-63640-9.00027-8

[27] Englot DJ, Han SJ, Lawton MT, Chang EF (2011) Predictors of seizure freedom in the surgical treatment of supratentorial cavernous malformations. J Neurosurg 115:1169–1174. 10.3171/2011.7.Jns11536 21819194 10.3171/2011.7.JNS11536

[28] Ferroli P, Casazza M, Marras C, Mendola C, Franzini A, Broggi G (2006) Cerebral cavernomas and seizures: a retrospective study on 163 patients who underwent pure lesionectomy. Neurol Sci 26:390–394. 10.1007/s10072-006-0521-2 16601930 10.1007/s10072-006-0521-2

[29] Fontanella MM, Bacigaluppi S, Doglietto F, Zanin L, Agosti E, Panciani P, Belotti F, Saraceno G, Spena G, Draghi R, Fiorindi A, Cornali C, Biroli A, Kivelev J, Chiesa M, Retta SF, Gasparotti R, Kato Y, Hernesniemi J, Rigamonti D (2021) An international call for a new grading system for cerebral and cerebellar cavernomas. J Neurosurg Sci 65:239–246. 10.23736/s0390-5616.21.05433-3 34184861 10.23736/S0390-5616.21.05433-3

[30] Fotakopoulos G, Kivelev J, Andrade-Barazarte H, Tjahjadi M, Goehre F, Hernesniemi J (2021) Outcome in patients with spinal cavernomas presenting with symptoms due to mass effect and/or hemorrhage: conservative versus surgical management: meta-analysis of direct comparison of approach-related complications. World Neurosurg 152:6–18. 10.1016/j.wneu.2021.05.094 34062296 10.1016/j.wneu.2021.05.094

[31] Garcia RM, Ivan ME, Lawton MT (2015) Brainstem cavernous malformations: surgical results in 104 patients and a proposed grading system to predict neurological outcomes. Neurosurgery 76:265–277. 10.1227/neu.0000000000000602. (discussion 277-268) 25599205 10.1227/NEU.0000000000000602

[32] Gibson CC, Zhu W, Davis CT, Bowman-Kirigin JA, Chan AC, Ling J, Walker AE, Goitre L, Delle Monache S, Retta SF, Shiu YT, Grossmann AH, Thomas KR, Donato AJ, Lesniewski LA, Whitehead KJ, Li DY (2015) Strategy for identifying repurposed drugs for the treatment of cerebral cavernous malformation. Circulation 131:289–299. 10.1161/circulationaha.114.010403 25486933 10.1161/CIRCULATIONAHA.114.010403 PMC4356181

[33] Girard R, Li Y, Stadnik A, Shenkar R, Hobson N, Romanos S, Srinath A, Moore T, Lightle R, Shkoukani A, Akers A, Carroll T, Christoforidis GA, Koenig JI, Lee C, Piedad K, Greenberg SM, Kim H, Flemming KD, Ji Y, Awad IA (2021) A roadmap for developing plasma diagnostic and prognostic biomarkers of cerebral cavernous angioma with symptomatic hemorrhage (CASH). Neurosurgery 88:686–697. 10.1093/neuros/nyaa478 33469662 10.1093/neuros/nyaa478 PMC7884145

[34] Girard R, Zeineddine HA, Koskimäki J, Fam MD, Cao Y, Shi C, Moore T, Lightle R, Stadnik A, Chaudagar K, Polster S, Shenkar R, Duggan R, Leclerc D, Whitehead KJ, Li DY, Awad IA (2018) Plasma biomarkers of inflammation and angiogenesis predict cerebral cavernous malformation symptomatic hemorrhage or lesional growth. Circ Res 122:1716–1721. 10.1161/circresaha.118.312680 29720384 10.1161/CIRCRESAHA.118.312680 PMC5993629

[35] Gomez-Paz S, Salem MM, Maragkos GA, Ascanio LC, Enriquez-Marulanda A, Lee M, Kicielinski KP, Moore JM, Thomas AJ, Ogilvy CS (2020) Role of aspirin and statin therapy in patients with cerebral cavernous malformations. J Clin Neurosci 78:246–251. 10.1016/j.jocn.2020.04.012 32340842 10.1016/j.jocn.2020.04.012

[36] Gross BA, Du R, Popp AJ, Day AL (2010) Intramedullary spinal cord cavernous malformations. Neurosurg Focus 29:E14. 10.3171/2010.6.Focus10144 20809755 10.3171/2010.6.FOCUS10144

[37] Hendricks BK, Rumalla K, Benner D, Lawton MT (2022) Cavernous malformations and artificial intelligence: machine learning applications. Neurosurg Clin N Am 33:461–467. 10.1016/j.nec.2022.05.007 36229133 10.1016/j.nec.2022.05.007

[38] Hendricks BK, Scherschinski L, Jubran JH, Dadario NB, Karahalios K, Benner D, VanBrabant D, Lawton MT (2024) Eloquent noneloquence: redefinition of cortical eloquence based on outcomes of superficial cerebral cavernous malformation resection. J Neurosurg 141:291–305. 10.3171/2023.12.Jns232588 38457787 10.3171/2023.12.JNS232588

[39] Herten A, Chen B, Saban D, Santos A, Wrede K, Jabbarli R, Zhu Y, Schmidt B, Kleinschnitz C, Forsting M, Sure U, Dammann P (2021) Health-related quality of life in patients with untreated cavernous malformations of the central nervous system. Eur J Neurol 28:491–499. 10.1111/ene.14546 32961598 10.1111/ene.14546

[40] Hoffman JE, Wittenberg B, Morel B, Folzenlogen Z, Case D, Roark C, Youssef S, Seinfeld J (2022) Tailored treatment options for cerebral cavernous malformations. J Pers Med 12. 10.3390/jpm12050831 10.3390/jpm12050831 PMC9147523 35629253

[41] Horne MA, Flemming KD, Su IC, Stapf C, Jeon JP, Li D, Maxwell SS, White P, Christianson TJ, Agid R, Cho WS, Oh CW, Wu Z, Zhang JT, Kim JE, Ter Brugge K, Willinsky R, Brown RD Jr, Murray GD, Al-Shahi Salman R (2016) Clinical course of untreated cerebral cavernous malformations: a meta-analysis of individual patient data. Lancet Neurol 15:166–173. 10.1016/s1474-4422(15)00303-8 10.1016/S1474-4422(15)00303-8 PMC4710581 26654287

[42] Ikramuddin S, Liu S, Ryan D, Hassani S, Hasan D, Feng W (2023) Propranolol or beta-blockers for cerebral cavernous malformation: a systematic review and meta-analysis of literature in both preclinical and clinical studies. Transl Stroke Res. 10.1007/s12975-023-01199-5 37857790 10.1007/s12975-023-01199-5

[43] Imagama S, Ito Z, Ando K, Kobayashi K, Hida T, Ito K, Tsushima M, Ishikawa Y, Matsumoto A, Morozumi M, Tanaka S, Machino M, Ota K, Nakashima H, Wakao N, Sakai Y, Matsuyama Y, Ishiguro N (2017) Optimal timing of surgery for intramedullary cavernous hemangioma of the spinal cord in relation to preoperative motor paresis, disease duration, and tumor volume and location. Glob Spine J 7:246–253. 10.1177/2192568217707938 10.1177/2192568217707938 PMC5476360 28660107

[44] Jabal MS, Mohammed MA, Kobeissi H, Lanzino G, Brinjikji W, Flemming KD (2024) Quantitative image signature and machine learning-based prediction of outcomes in cerebral cavernous malformations. J Stroke Cerebrovasc Dis 33:107462. 10.1016/j.jstrokecerebrovasdis.2023.107462 37931483 10.1016/j.jstrokecerebrovasdis.2023.107462

[45] Kearns KN, Chen CJ, Tvrdik P, Park MS, Kalani MYS (2019) Outcomes of surgery for brainstem cavernous malformations: a systematic review. Stroke 50:2964–2966. 10.1161/strokeaha.119.026120 31510895 10.1161/STROKEAHA.119.026120

[46] Kivelev J, Niemelä M, Hernesniemi J (2012) Treatment strategies in cavernomas of the brain and spine. J Clin Neurosci 19:491–497. 10.1016/j.jocn.2011.08.015 22325075 10.1016/j.jocn.2011.08.015

[47] La Rocca G, Ius T, Mazzucchi E, Simboli GA, Altieri R, Garbossa D, Acampora A, Auricchio AM, Vincitorio F, Cofano F, Vercelli G, Della Pepa GM, Pignotti F, Albanese A, Marchese E, Sabatino G (2020) Trans-sulcal versus trans-parenchymal approach in supratentorial cavernomas. A multicentric experience. Clin Neurol Neurosurg 197:106180. 10.1016/j.clineuro.2020.106180 32877767 10.1016/j.clineuro.2020.106180

[48] Lanfranconi S, Scola E, Meessen J, Pallini R, Bertani GA, Al-Shahi Salman R, Dejana E, Latini R (2023) Safety and efficacy of propranolol for treatment of familial cerebral cavernous malformations (Treat_CCM): a randomised, open-label, blinded-endpoint, phase 2 pilot trial. Lancet Neurol 22:35–44. 10.1016/s1474-4422(22)00409-4 36403580 10.1016/S1474-4422(22)00409-4

[49] Lawton MT, Lang MJ (2019) The future of open vascular neurosurgery: perspectives on cavernous malformations, AVMs, and bypasses for complex aneurysms. J Neurosurg 130:1409–1425. 10.3171/2019.1.Jns182156 31042667 10.3171/2019.1.JNS182156

[50] Lazzaroni F, Meessen J, Sun Y, Lanfranconi S, Scola E, D’Alessandris QG, Tassi L, Carriero MR, Castori M, Marino S, Blanda A, Nicolis EB, Novelli D, Calabrese R, Agnelli NM, Bottazzi B, Leone R, Mazzola S, Besana S, Catozzi C, Nezi L, Lampugnani MG, Malinverno M, Grdseloff N, Rödel CJ, Rezai Jahromi B, Bolli N, Passamonti F, Magnusson PU, Abdelilah-Seyfried S, Dejana E, Latini R (2024) Circulating biomarkers in familial cerebral cavernous malformation. EBioMedicine 99:104914. 10.1016/j.ebiom.2023.104914 38113759 10.1016/j.ebiom.2023.104914 PMC10767159

[51] Li X, Zhang H, Ren J (2024) Intraoperative changes in electrophysiological monitoring can be used to predict clinical outcomes in patients with spinal cavernous malformation. Open Med (Wars) 19:20241008. 10.1515/med-2024-1008 39434862 10.1515/med-2024-1008 PMC11491772

[52] Louis DN, Perry A, Wesseling P, Brat DJ, Cree IA, Figarella-Branger D, Hawkins C, Ng HK, Pfister SM, Reifenberger G, Soffietti R, von Deimling A, Ellison DW (2021) The 2021 WHO classification of tumors of the central nervous system: a summary. Neuro Oncol 23:1231–1251. 10.1093/neuonc/noab106 34185076 10.1093/neuonc/noab106 PMC8328013

[53] Malcolm JG, Douglas JM, Greven A, Rich C, Dawoud RA, Hu R, Reisner A, Barrow DL, Gross RE, Willie JT (2021) Feasibility and morbidity of magnetic resonance imaging-guided stereotactic laser ablation of deep cerebral cavernous malformations: a report of 4 cases. Neurosurgery 89:635–644. 10.1093/neuros/nyab241 34270738 10.1093/neuros/nyab241

[54] Marotta D, Hendricks BK, Zaher M, Watanabe G, Grasso G, Cohen-Gadol A (2022) Resection of brainstem cavernous malformations: pearls and pitfalls for minimizing complications. World Neurosurg 159:390–401. 10.1016/j.wneu.2021.08.072 35255638 10.1016/j.wneu.2021.08.072

[55] Marques LL, Jaeggi C, Branca M, Raabe A, Bervini D, Goldberg J (2023) Bleeding risk of cerebral cavernous malformations in patients on statin and antiplatelet medication: a cohort study. Neurosurgery 93:699–705. 10.1227/neu.0000000000002480 36999926 10.1227/neu.0000000000002480

[56] Mooney MA, Zabramski JM (2017) Developmental venous anomalies. Handb Clin Neurol 143:279–282. 10.1016/b978-0-444-63640-9.00026-6 28552150 10.1016/B978-0-444-63640-9.00026-6

[57] Morrison L, Gutierrez J, Ayata C, Lopez-Toledano M, Carrazana E, Awad I, Rabinowicz AL, Kim H (2024) Current and future treatment options for cerebral cavernous malformations. Stroke: Vasc Interv Neurol 4:e001140. 10.1161/SVIN.123.001140 10.1212/NXG.0000000000200121 PMC10766084 38179414

[58] Moultrie F, Horne MA, Josephson CB, Hall JM, Counsell CE, Bhattacharya JJ, Papanastassiou V, Sellar RJ, Warlow CP, Murray GD, Al-Shahi Salman R (2014) Outcome after surgical or conservative management of cerebral cavernous malformations. Neurology 83:582–589. 10.1212/wnl.0000000000000684 24994841 10.1212/WNL.0000000000000684 PMC4141991

[59] Musmar B, Salim H, Abdelgadir J, Spellicy S, Adeeb N, Zomorodi A, Friedman A, Awad I, Jabbour PM, Hasan DM (2024) Antithrombotic therapy in cerebral cavernous malformations: a systematic review, meta-analysis, and network meta-analysis. J Am Heart Assoc 13:e032910. 10.1161/jaha.123.032910 38471833 10.1161/JAHA.123.032910 PMC11010038

[60] Paiva WS, Fonoff ET, Marcolin MA, Bor-Seng-Shu E, Figueiredo EG, Teixeira MJ (2013) Navigated transcranial magnetic stimulation in preoperative planning for the treatment of motor area cavernous angiomas. Neuropsychiatr Dis Treat 9:1885–1888. 10.2147/ndt.S43644 24353424 10.2147/NDT.S43644 PMC3862695

[61] Pamias-Portalatin E, Duran IS, Ebot J, Bojaxhi E, Tatum W, Quiñones-Hinojosa A (2018) Awake-craniotomy for cavernoma resection. Neurosurg Focus 45:V3. 10.3171/2018.10.FocusVid.18201 30269557 10.3171/2018.10.FocusVid.18201

[62] Petersen TA, Morrison LA, Schrader RM, Hart BL (2010) Familial versus sporadic cavernous malformations: differences in developmental venous anomaly association and lesion phenotype. AJNR Am J Neuroradiol 31:377–382. 10.3174/ajnr.A1822 19833796 10.3174/ajnr.A1822 PMC4455949

[63] Poorthuis M, Samarasekera N, Kontoh K, Stuart I, Cope B, Kitchen N, Al-Shahi Salman R (2013) Comparative studies of the diagnosis and treatment of cerebral cavernous malformations in adults: systematic review. Acta Neurochir (Wien) 155:643–649. 10.1007/s00701-013-1621-4 23371401 10.1007/s00701-013-1621-4

[64] Poorthuis MHF, Rinkel LA, Lammy S, Al-Shahi Salman R (2019) Stereotactic radiosurgery for cerebral cavernous malformations: a systematic review. Neurology 93:e1971–e1979. 10.1212/wnl.0000000000008521 31659093 10.1212/WNL.0000000000008521

[65] Rauschenbach L, Bartsch P, Santos AN, Lenkeit A, Darkwah Oppong M, Wrede KH, Jabbarli R, Chmielewski WX, Schmidt B, Quesada CM, Forsting M, Sure U, Dammann P (2022) Quality of life and mood assessment in conservatively treated cavernous malformation-related epilepsy. Brain Behav 12:e2595. 10.1002/brb3.2595 35470577 10.1002/brb3.2595 PMC9226805

[66] Rauschenbach L, Santos AN, Dinger TF, Herten A, Darkwah Oppong M, Schmidt B, Chihi M, Haubold J, Jabbarli R, Wrede KH, Sure U, Dammann P (2021) Predictive value of intraoperative neuromonitoring in brainstem cavernous malformation surgery. World Neurosurg 156:e359–e373. 10.1016/j.wneu.2021.09.064 34560298 10.1016/j.wneu.2021.09.064

[67] Rauschenbach L, Santos AN, Gull HH, Rieß C, Deuschl C, Schmidt B, Darkwah Oppong M, Gembruch O, Özkan N, Jabbarli R, Wrede KH, Sure U, Dammann P (2023) Functional impact of multiple bleeding events in patients with conservatively treated spinal cavernous malformations. J Neurosurg Spine 38:405–411. 10.3171/2022.10.Spine22940 36401548 10.3171/2022.10.SPINE22940

[68] Ren J, Hong T, He C, Li X, Ma Y, Yu J, Ling F, Zhang H (2019) Surgical approaches and long-term outcomes of intramedullary spinal cord cavernous malformations: a single-center consecutive series of 219 patients. J Neurosurg Spine 31:123–132. 10.3171/2018.12.Spine181263 30952112 10.3171/2018.12.SPINE181263

[69] Rinkel GJE (2024) Cerebral cavernous malformations: to operate or not? Lancet Neurol 23:546–547. 10.1016/s1474-4422(24)00161-3 38760083 10.1016/S1474-4422(24)00161-3

[70] Rosenow F, Alonso-Vanegas MA, Baumgartner C, Blümcke I, Carreño M, Gizewski ER, Hamer HM, Knake S, Kahane P, Lüders HO, Mathern GW, Menzler K, Miller J, Otsuki T, Ozkara C, Pitkänen A, Roper SN, Sakamoto AC, Sure U, Walker MC, Steinhoff BJ (2013) Cavernoma-related epilepsy: review and recommendations for management–report of the Surgical Task Force of the ILAE Commission on Therapeutic Strategies. Epilepsia 54:2025–2035. 10.1111/epi.12402 24134485 10.1111/epi.12402

[71] Ruan D, Yu XB, Shrestha S, Wang L, Chen G (2015) The role of hemosiderin excision in seizure outcome in cerebral cavernous malformation surgery: a systematic review and meta-analysis. PLoS ONE 10:e0136619. 10.1371/journal.pone.0136619 26305879 10.1371/journal.pone.0136619 PMC4548944

[72] Rumalla K, Srinivasan VM, Gaddis M, Kvint S, Patel AJ, Kan P, Lawton MT, Burkhardt JK (2021) Cavernous malformation surgery in the United States: validation of a novel international classification of disease, 10th edition, clinical modification code search algorithm and volume-driven surgical outcomes. World Neurosurg 150:e66–e73. 10.1016/j.wneu.2021.02.081 33640531 10.1016/j.wneu.2021.02.081

[73] Santos AN, Rauschenbach L, Riess C, Georgiades I, Fiçilar B, Gallardo EG, Quesada CM, Li Y, Tippelt S, Dohna-Schwake C, Schmidt B, Jabbarli R, Siegel AM, Benet A, Wrede KH, Sure U, Dammann P (2023) Outcome after conservative or surgical treatment for new-onset epilepsy in children with cerebral cavernous malformation. Seizure 111:23–29. 10.1016/j.seizure.2023.07.011 37494759 10.1016/j.seizure.2023.07.011

[74] Santos AN, Rauschenbach L, Saban D, Chen B, Darkwah Oppong M, Herten A, Gull HH, Rieß C, Deuschl C, Schmidt B, Jabbarli R, Wrede KH, Zhu Y, Frank B, Sure U, Dammann P (2022) Multiple cerebral cavernous malformations: clinical course of confirmed, assumed and non-familial disease. Eur J Neurol 29:1427–1434. 10.1111/ene.15253 35060255 10.1111/ene.15253

[75] Sevy A, Gavaret M, Trebuchon A, Vaugier L, Wendling F, Carron R, Regis J, Chauvel P, Gonigal AM, Bartolomei F (2014) Beyond the lesion: the epileptogenic networks around cavernous angiomas. Epilepsy Res 108:701–708. 10.1016/j.eplepsyres.2014.02.018 24661427 10.1016/j.eplepsyres.2014.02.018

[76] Shenkar R, Shi C, Rebeiz T, Stockton RA, McDonald DA, Mikati AG, Zhang L, Austin C, Akers AL, Gallione CJ, Rorrer A, Gunel M, Min W, De Souza JM, Lee C, Marchuk DA, Awad IA (2015) Exceptional aggressiveness of cerebral cavernous malformation disease associated with PDCD10 mutations. Genet Med 17:188–196. 10.1038/gim.2014.97 25122144 10.1038/gim.2014.97 PMC4329119

[77] Shoubash L, Baldauf J, Matthes M, Kirsch M, Rath M, Felbor U, Schroeder HWS (2022) Long-term outcome and quality of life after CNS cavernoma resection: eloquent vs. non-eloquent areas. Neurosurg Rev 45:649–660. 10.1007/s10143-021-01572-8 34164745 10.1007/s10143-021-01572-8 PMC8827309

[78] Snellings DA, Hong CC, Ren AA, Lopez-Ramirez MA, Girard R, Srinath A, Marchuk DA, Ginsberg MH, Awad IA, Kahn ML (2021) Cerebral cavernous malformation: from mechanism to therapy. Circ Res 129:195–215. 10.1161/circresaha.121.318174 34166073 10.1161/CIRCRESAHA.121.318174 PMC8922476

[79] Srinath A, Xie B, Li Y, Sone JY, Romanos S, Chen C, Sharma A, Polster S, Dorrestein PC, Weldon KC, DeBiasse D, Moore T, Lightle R, Koskimäki J, Zhang D, Stadnik A, Piedad K, Hagan M, Shkoukani A, Carrión-Penagos J, Bi D, Shen L, Shenkar R, Ji Y, Sidebottom A, Pamer E, Gilbert JA, Kahn ML, D’Souza M, Sulakhe D, Awad IA, Girard R (2023) Plasma metabolites with mechanistic and clinical links to the neurovascular disease cavernous angioma. Commun Med (Lond) 3:35. 10.1038/s43856-023-00265-1 36869161 10.1038/s43856-023-00265-1 PMC9984539

[80] Stapleton CJ, Barker FG 2nd (2018) Cranial cavernous malformations: natural history and treatment. Stroke 49:1029–1035. 10.1161/strokeaha.117.017074 29535273 10.1161/STROKEAHA.117.017074

[81] Tasiou A, Brotis AG, Kalogeras A, Tzerefos C, Alleyne CH Jr, Andreou A, Demetriades AK, Foroglou N, Friedlander RM, Karlsson B, Kitchen N, Meling TR, Mitsos A, Panagiotopoulos V, Papasilekas T, Pavesi G, Rasulic L, Santos AN, Spetzler RF, Sure U, Tjoumakaris S, Tolias CM, Vajkoczy P, Fountas KN (2023) Cavernous malformations of the central nervous system: an international consensus statement. Brain Spine 3:102707. 10.1016/j.bas.2023.102707 38020995 10.1016/j.bas.2023.102707 PMC10668094

[82] Walicke P, Abosch A, Asher A, Barker FG 2nd, Ghogawala Z, Harbaugh R, Jehi L, Kestle J, Koroshetz W, Little R, Rubin D, Valadka A, Wisniewski S, Chiocca EA (2017) Launching effectiveness research to guide practice in neurosurgery: a National Institute Neurological Disorders and Stroke Workshop Report. Neurosurgery 80:505–514. 10.1093/neuros/nyw133 28362926 10.1093/neuros/nyw133 PMC5808147

[83] Wildi S, Nager S, Akeret K, Özkaratufan S, Krayenbühl N, Bozinov O, Regli L, Velz J (2023) Impact of long-term antithrombotic and statin therapy on the clinical outcome in patients with cavernous malformations of the central nervous system: a single-center case series of 428 patients. Cerebrovasc Dis 52:634–642. 10.1159/000529511 36944322 10.1159/000529511 PMC10906472

[84] Yadla S, Jabbour PM, Shenkar R, Shi C, Campbell PG, Awad IA (2010) Cerebral cavernous malformations as a disease of vascular permeability: from bench to bedside with caution. Neurosurg Focus 29:E4. 10.3171/2010.5.Focus10121 20809762 10.3171/2010.5.FOCUS10121 PMC6599630

[85] Yang Y, Velz J, Neidert MC, Lang W, Regli L, Bozinov O (2022) The BSCM score: a guideline for surgical decision-making for brainstem cavernous malformations. Neurosurg Rev 45:1579–1587. 10.1007/s10143-021-01679-y 34713352 10.1007/s10143-021-01679-y PMC8976795

[86] Yu T, Liu X, Lin X, Bai C, Zhao J, Zhang J, Zhang L, Wu Z, Wang S, Zhao Y, Meng G (2016) The relation between angioarchitectural factors of developmental venous anomaly and concomitant sporadic cavernous malformation. BMC Neurol 16:183. 10.1186/s12883-016-0691-3 27660100 10.1186/s12883-016-0691-3 PMC5034432

[87] Zanello M, Meyer B, Still M, Goodden JR, Colle H, Schichor C, Bello L, Wager M, Smits A, Rydenhag B, Tate M, Metellus P, Hamer PW, Spena G, Capelle L, Mandonnet E, Robles SG, Sarubbo S, Martino González J, Fontaine D, Reyns N, Krieg SM, Huberfeld G, Wostrack M, Colle D, Robert E, Noens B, Muller P, Yusupov N, Rossi M, Conti Nibali M, Papagno C, Visser V, Baaijen H, Galbarritu L, Chioffi F, Bucheli C, Roux A, Dezamis E, Duffau H, Pallud J (2019) Surgical resection of cavernous angioma located within eloquent brain areas: international survey of the practical management among 19 specialized centers. Seizure 69:31–40. 10.1016/j.seizure.2019.03.022 30959423 10.1016/j.seizure.2019.03.022

[88] Zhang P, Liu L, Cao Y, Wang S, Zhao J (2013) Cerebellar cavernous malformations with and without associated developmental venous anomalies. BMC Neurol 13:134. 10.1186/1471-2377-13-134 24088363 10.1186/1471-2377-13-134 PMC3850546

[89] Zhao J, Wang Y, Kang S, Wang S, Wang J, Wang R, Zhao Y (2007) The benefit of neuronavigation for the treatment of patients with intracerebral cavernous malformations. Neurosurg Rev 30:313–318. 10.1007/s10143-007-0080-x. (discussion 319) 17629759 10.1007/s10143-007-0080-x

[90] Zotta D, Di Rienzo A, Scogna A, Ricci A, Ricci G, Galzio RJ (2005) Supratentorial cavernomas in eloquent brain areas: application of neuronavigation and functional MRI in operative planning. J Neurosurg Sci 49:13–19 15990714

[91] Zuurbier SM, Hickman CR, Tolias CS, Rinkel LA, Leyrer R, Flemming KD, Bervini D, Lanzino G, Wityk RJ, Schneble HM, Sure U, Al-Shahi Salman R (2019) Long-term antithrombotic therapy and risk of intracranial haemorrhage from cerebral cavernous malformations: a population-based cohort study, systematic review, and meta-analysis. Lancet Neurol 18:935–941. 10.1016/s1474-4422(19)30231-5 31401075 10.1016/S1474-4422(19)30231-5 PMC6744367

[92] Zuurbier SM, Santos AN, Flemming KD, Schmidt B, Jabbarli R, Lanzino G, Sure U, Dammann P (2023) Female hormone therapy and risk of intracranial hemorrhage from cerebral cavernous malformations: a multicenter observational cohort study. Neurology 100:e1673–e1679. 10.1212/wnl.0000000000206888 36754635 10.1212/WNL.0000000000206888 PMC10115495

